# Enhancing Antioxidants Performance of Ceria Nanoparticles in Biological Environment via Surface Engineering with *o-*Quinone Functionalities

**DOI:** 10.3390/antiox14080916

**Published:** 2025-07-25

**Authors:** Pierluigi Lasala, Tiziana Latronico, Umberto Mattia, Rosa Maria Matteucci, Antonella Milella, Matteo Grattieri, Grazia Maria Liuzzi, Giuseppe Petrosillo, Annamaria Panniello, Nicoletta Depalo, Maria Lucia Curri, Elisabetta Fanizza

**Affiliations:** 1 Chemistry Department, University of Bari, Via Orabona 4, 70126 Bari, Italy; pierluigi.lasala@uniba.it (P.L.); u.mattia@studenti.uniba.it (U.M.); antonella.milella@uniba.it (A.M.); matteo.grattieri@uniba.it (M.G.); marialucia.curri@uniba.it (M.L.C.); 2 Department of Bioscience, Biotechnology and Environment, University of Bari, Via Orabona 4, 70126 Bari, Italy; tiziana.latronico@uniba.it (T.L.); graziamaria.liuzzi@uniba.it (G.M.L.); 3 Department of Electrical and Information Engineering, Polytechnic University of Bari, Via Orabona 4, 70125 Bari, Italy; r.matteucci@phd.poliba.it; 4 CNR—IPCF Institute for Chemical-Physical Process, Via Orabona 4, 70126 Bari, Italy; annamaria.panniello@cnr.it (A.P.); nicoletta.depalo@cnr.it (N.D.); 5 National Interuniversity Consortium of Materials Science and Technology, INSTM, Bari Research Unit, 70126 Bari, Italy; 6 CNR—IBIOM Institute of Biomembranes, Bioenergetics and Molecular Biotechnologies, Via Giovanni Amendola, 122/O, 70126 Bari, Italy; giuseppe.petrosillo@cnr.it

**Keywords:** ceria nanoparticles, non-enzymatic assay for antioxidant studies, autoxidation chain-breaking, alkylperoxyl and hydroxyperoxyl radicals

## Abstract

The development of ceria (CeO_2−x_)-based nanoantioxidants requires fine-tuning of structural and surface properties for enhancing antioxidant behavior in biological environments. In this contest, here ultrasmall water-dispersible CeO_2−x_ nanoparticles (NPs), characterized by a high Ce^3+^/Ce^4+^ ratio, were synthesized in a non-polar solvent and phase-transfer to an aqueous environment through ligand-exchange reactions using citric acid (CeO_2−x_@Cit) and post-treatment with dopamine hydrochloride (CeO_2−x_@Dopa). The concept behind this work is to enhance via surface engineering the intrinsic antioxidant properties of CeO_2−x_ NPs. For this purpose, thanks to electron transfer reactions between dopamine and CeO_2−x_, the CeO_2−x_@Dopa was obtained, characterized by increased surface Ce^3+^ sites and surface functionalized with polydopamine bearing *o*-quinone structures as demonstrated by complementary spectroscopic (UV–vis, FT-IR, and XPS) characterizations. To test the antioxidant properties of CeO_2−x_ NPs, the scavenging activity before and after dopamine treatment against artificial radical 1,1-diphenyl-2-picrylhydrazyl (DPPH^·^) and the ability to reduce the reactive oxygen species in Diencephalic Immortalized Type Neural Cell line 1 were evaluated. CeO_2−x_@Dopa demonstrated less efficiency in DPPH^·^ scavenging (%radical scavenging activity 13% versus 42% for CeO_2−x_@Cit before dopamine treatment at 33 μM DPPH^·^ and 0.13 mg/mL loading of NPs), while it markedly reduced intracellular ROS levels (ROS production 35% compared to 66% of CeO_2−x_@Cit before dopamine treatment with respect to control—*p* < 0.001 and *p* < 0.01, respectively). While steric hindrance from the dopamine-derived polymer layer limited direct electron transfer from CeO_2−x_ NP surface to DPPH^·^, within cells the presence of *o*-quinone groups contributed with CeO_2−x_ NPs to break the autoxidation chain of organic substrates, enhancing the antioxidant activity. The functionalization of NPs with *o*-quinone structures represents a valuable approach to increase the inherent antioxidant properties of CeO_2−x_ NPs, enhancing their effectiveness in biological systems by promoting additional redox pathways.

## 1. Introduction

Nanoceria is commonly referred to CeO_2−x_ [1] to reflect the significant presence of Ce^3+^ sites alongside Ce^4+^, compared to its bulk counterpart. In small NPs, oxygen loss from the crystal lattice—due to surface strain—leads to the formation of oxygen vacancies accompanied by the concurrent reduction of Ce^4+^ to Ce^3+^ [2,3,4,5]. The coexistence of the two valence states in CeO_2−x_ NPs, and the low redox potential of the Ce^4+^/Ce^3+^ couple^,^ in nanosized particles that make easy the shift between Ce^3+^ and Ce^4+^ determine the intrinsic antioxidant properties within cells and tissues [6]. Concomitantly, it is important to note that CeO_2−x_ NPs can also act as pro-oxidants, particularly under acidic conditions [7], and recent studies highlighted the ability to produce oxidative stress under acidic conditions. However, under physiologic pH antioxidant activity is generally favored [8].

The antioxidant behavior and their widely recognized role as nanozymes are due to CeO_2−x_ NPs ability to scavenge reactive oxygen species (ROS), such as superoxide (O_2_^−^) and hydrogen peroxide (H_2_O_2_), which, in general, are physiologically degraded by the redox enzymes superoxide dismutase (SOD) and catalase (CAT) [9,10,11].

**Figure 1:**
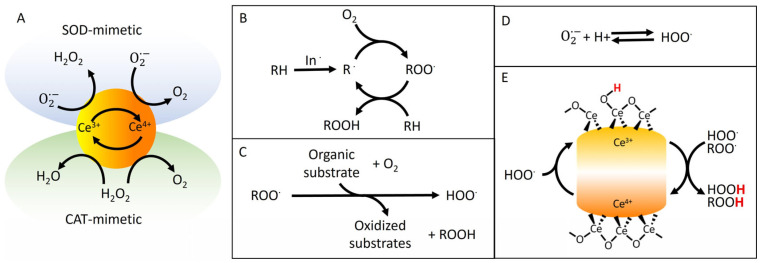
(**A**) Sketch of superoxide dismutase and catalase mimetic activity of CeO_2−x_ NPs. (**B**) Scheme of the autoxidation chain of organic substrates, contributing to the release of hydroperoxyl radical (HOO^·^, (**C**)) also produced by protonation of superoxide anion radical (**D**). Role of CeO_2−x_ NPs in chain breaking autoxidation processes (**E**).

In biological environments, the NPs SOD-like activity (Figre1A) can attenuate the production of ^·^O_2_^−^, which is transformed into O_2_ and H_2_O_2_. Concomitantly, the CAT-like activity (Figure 1A) can remove the H_2_O_2_ disproportionated into H_2_O and O_2_. Theoretical studies suggest that, under physiological pH, the direct reduction of ^·^O_2_^−^ to oxygen is not feasible and only the conversion to H_2_O_2_ occurs. Similarly, the CAT-like activity only proceeds through H_2_O_2_ adsorption onto the NP surface, forming a cerium-oxo-peroxo complex that subsequently dissociates to release O_2_. Therefore, CeO_2−x_ NPs can reversibly regenerate their oxidation state, shifting between Ce^3+^ and Ce^4+^, by interacting sequentially with ^·^O_2_^−^ and H_2_O_2_ species.

Interestingly, CeO_2−x_ NPs exhibit antioxidant properties beyond these enzyme-like mechanisms. Recent findings indicate that they also catalyze additional redox reactions, including chain breaking of organic substrate autoxidation (e.g., lipid peroxidation) and quenching of hydroperoxyl radical HOO^·^ [6,12], both strongly contributing to the propagation of oxidative stress and damage of biomolecules [13]. In the autoxidation pathway (Figure 1B), alkyl radicals, R^·^, generated by the reaction of bioorganic compounds with radical initiators, readily react with O_2_ and convert into alkylperoxyl ROO^·^ radicals. A cyclic reaction involving RH species contributes to the propagation of the organic substrate autooxidation [12]. Amorati et al. [6,12,14] demonstrated that CeO_2−x_ NPs can break the autoxidation of organic substrate, competing with RH (Figure 1D, right side), with Ce(III)-OH sites giving a proton and electron to ROO^·^, forming ROOH while oxidizing to Ce(IV)[14]. Furthermore, CeO_2−x_ NPs can also effectively quench HOO^·^ [14], the protonated form of ^·^O_2_^−^, which is produced either under acidic conditions (pKa = 4.8) (Figure 1D) or generated by the autoxidation of various organic substrates (Figure 1C).

While acidic pH conditions are uncommon under physiological conditions, specific membrane domains may sustain the formation of HOO^·^. CeO_2−x_ NPs can quench HOO^·^ by taking advantage of its reducing and oxidizing power, wherein HOO^·^ radicals donate an electron and a proton to Ce(IV) surface sites, generating Ce(III)-OH and releasing O_2_ (Figure 1E left side). This Ce(III)-OH can subsequently regenerate Ce(IV) by further reacting with ROO· or HOO^·^. These diverse antioxidant mechanisms are closely related to the Ce^3+^/Ce^4+^ ratio within the NPs. Several literature studies have highlighted the superior ability of Ce^3+^ to engage in redox reactions compared to Ce^4+^, facilitating more effective neutralization of ROS [15] and, thus, enhancement of the antioxidant activities [4]. The Ce^3+^ content is highly dependent on NP size and surface chemistry. As the size of the CeO_2−x_ NPs decreases, NPs undergo an increase in Ce^3+^/Ce^4+^ ratio [16,17], which may promote improved antioxidant properties [18]. Furthermore, rich surface chemistry, including surface hydroxylation and surface functionalization, can significantly affect redox behavior and hence catalytic activities of CeO_2−x_ NPs. Modifications of ceria surface states due to molecule adsorption or anchoring can increase or decrease the antioxidant properties of CeO_2−x_ NPs depending on the electronic properties and density of the functionalization [19]. Polymers or co-polymer coatings [20,21], such as polyethylene glycol or polyacrylic acid, have been explored to impart good colloidal stability, which enhances surface availability and hence catalytic reaction, while inhibiting protein adsorption in biological media. However, the nature and the thickness of the polymer coating can variably impact the enzyme-like activity of CeO_2−x_ NPs. While some reports show minimal effects on CAT- and SOD-like functions, others note suppression of CAT and oxidase mimetic activity, with a concurrent increase in peroxidase-like activity. Lee et al. [22] demonstrated that thicker polymer coatings can diminish intracellular and in vitro radical scavenging due to reduced accessibility of redox active sites. Surface functionalization with polymers featuring reactive groups, such as catechols, has also been investigated. Hu et al. [16] functionalized CeO_2−x_ NPs with catechol-grafted poly(ethylene)glycol. The interaction of CeO_2−x_ NPs with catechol functionalities, studied in the last decade using biologically relevant catechol-containing molecules [23], such as dopamine [24], highlights that these molecules can form strong metal–ligand complexes by transferring electrons from the catechol group (oxidized to *o-*quinone) to Ce^4+^, reducing it to Ce^3+^, and thereby enhancing the antioxidant capacity of the NPs via increased Ce^3+^ content. However, the specific role of surface *o*-quinone functionalities in enhancing redox activity, and hence antioxidant behavior of the hybrid structure, remains underexplored. Recent studies by Amorati et al. [25] have demonstrated that *o*-quinone exhibits significant reactivity towards HOO^·^, effectively interrupting the autoxidation chain through concurrent scavenging of both HOO^·^ and ROO^.^ radicals.

Based on this insight, the aim of this study was to develop water-dispersible ultrasmall NPs CeO_2−x_ NPs with enhanced antioxidant properties through targeted surface engineering. Specifically, we employed a two- step strategy: (i) synthesis of ultrasmall hydrophobic ceria NPs via thermal decomposition of a cerium precursor in an organic solvent in the presence of oleyl amine, followed by (ii) surface modification through ligand exchange with citric acid (CeO_2−x_@Cit) to render them water dispersible, and subsequent post-functionalization using dopamine hydrochloride (CeO_2−x_@Dopa). The post-synthetic treatment was designed to promote Ce^3+^ surface enrichment and to introduce *o*-quinone functionalities on the NP surface and at the CeO_2−x_ NPs, via a metal–ligand electron transfer reaction with catechol or polydopamine moieties, which promote catalytic redox reactions that concomitantly quench HOO^·^ radicals, ultimately preventing the autoxidation chain propagation. Through a combination of spectroscopic and thermogravimetric analyses, the NPs surface properties have been depicted, and by testing CeO_2−x_ before and after dopamine treatment in scavenging DPPH^·^ and reacting with H_2_O_2_ as well as in a biological environment using Diencephalic Immortalized Type Neural Cell line 1 (DITNC1), superior intracellular ROS scavenging activity of CeO_2−x_@Dopa has been observed.

## 2. Materials and Methods

### 2.1. Materials

Cerium(III) nitrate hexahydrate (Ce(NO_3_)_3_·6H_2_O), oleylamine (OAm), oleic acid (ODE), toluene, methanol, isopropanol, acetone, hexane, chloroform, cyclohexane, citric acid, dopamine hydrochloride, ciclopentyl methyl ether (CME), 1,1-Diphenyl-2-picrylhydrazyl (DPPH), ammonium hydroxide (NH_4_OH), 2′,7′-Dichlorodihydrofluorescein diacetate (DCFH-DA, from Calbiochem), 3-(4,5-dimethylthiazol-2-yl)-2.5-diphenyltetrazolium bromide (MTT), ultrapure Milli-Q water (18 MΩ cm^–1^). All reagents were purchased from Sigma-Aldrich (St. Louis, MO, USA), if not differently stated. The DI-TNC1 cell line (ATCC CRL-2005) was acquired and authenticated from the ATCC (www.lgcstandards-atcc.org, accessed on 21 November 2024). All chemicals and solvents were of analytical grade and used as received without further purification.

### 2.2. Synthesis of Oleyl Amine-Capped CeO_2−x_ NPs (CeO_2−x_@OAm)

CeO_2−x_ NPs synthesis was carried out using a Schlenk line under nitrogen atmosphere and temperature conditions, following a procedure reported in literature [26,27,28] with minor modification. Briefly, 0.434 g (1 mmol) of Ce(NO_3_)_3_·6H_2_O was poured in a three-neck flask, and 2 mL (6 mmol) of OAm and 6 mL of ODE were added. The reaction mixture was first put under vacuum for 30 min at 80 °C under continuous stirring, obtaining a brown-colored solution, then nitrogen flowed into the flask and the temperature was increased up to 300 °C. The dark brown solution was left at this temperature for 60 min prior to being cooled down to room temperature. Once 160 °C was reached during the cooling ramp, 2 mL of toluene were injected into the reaction flask. NPs were finally collected, and unreacted reagents were removed by four cycles of addition of a large excess (10 times the reaction mixture volume) of isopropanol: acetone (1:1 volumetric ratio), followed by centrifugation at 1000 x g for 10 min. After discharging the supernatant, a few drops of hexane were added, followed by the addition of isopropanol/acetone and a centrifugation step to collect a black precipitate, which was finally dispersed in 5 mL of chloroform. An aliquot of the NPs solution was put under nitrogen flux to evaporate the solvent, and the remaining pellet was weighed to determine the NPs concentration, which resulted in about 56 mg/mL.

### 2.3. Phase Transfer of Organic-Capped CeO_2−x_ Nanoparticles in Aqueous Medium by Ligand-Exchange Reaction with Citrate (CeO_2−x_@Cit)

In order to replace OAm with citrate, a ligand-exchange procedure was developed by slightly modifying the Monnier et al. protocol [29]. Briefly, 5 mg of citric acid was dissolved in 1 mL of CME, then 100 μL of the CeO_2−x_@OAm NPs was added to the mixture and left to stir at 100 °C for 18 h. The NPs were then collected by centrifugation by adding methanol (16,000× *g* for 10 min). Two repeated cycles of dispersion in acetone and a centrifugation step at 16,000× *g* for 15 min were then carried out. The recovered pellet was redispersed in MilliQ H_2_O, where a few drops of NH_4_OH solution were added, resulting in a stable colloidal solution, which was stored at 4 °C for further use. An aliquot of the sample, labelled CeO_2−x_@Cit, was freeze-dried, resulting in a concentration of 44 mg/mL.

### 2.4. Functionalization of CeO_2−x_@Cit NPs with Dopamine (CeO_2−x_@Dopa)

A sample labelled CeO_2−x_@Dopa was prepared by adding dopamine hydrochloride to CeO_2−x_@Cit. In particular, 80 μL of 1.5 mM dopamine hydrochloride aqueous solution was added to 1 mL of 10 mg/mL of CeO_2−x_@Cit sample. Upon addition of dopamine hydrochloride, the NPs suspension turns brown. The solution was left stirring overnight at room temperature and finally collected after several cycles of purification by centrifugation and pellet dispersion in MilliQ water. An aliquot of the sample was freeze-dried, resulting in a concentration of 11 mg/mL.

### 2.5. Spectrophotometric Characterization of CeO_2−x_ Nanoparticles Treated with H_2_O_2_

The reactivity of CeO_2−x_@Cit or CeO_2−x_@Dopa towards H_2_O_2_ was spectrophotometrically evaluated. In a typical experiment, 1.5 mL of aqueous solution of NPs, either CeO_2−x_@Cit or CeO_2−x_@Dopa, at a concentration of 0.1 mg/mL was prepared, and 30 µL of H_2_O_2_ 50 mM was added ([H_2_O_2_] = 1 mM) and stirred for 30 min at room temperature. UV–vis absorption spectra of the colloidal solutions before and after H_2_O_2_ addition were recorded in the 200–800 nm wavelength range.

### 2.6. Spectrophotometric Analysis of 1,1-diphenyl-2-picrylhydrazil (DPPH^·^) Radical Scavenging by CeO_2−x_ Nanoparticles

The DPPH^·^ radical scavenging activity (RSA) was evaluated by monitoring the absorbance decrease at 517 nm of a DPPH^·^ solution at 0.033 mM in EtOH after it was left in the dark for 30 min with and without CeO_2−x_ NPs samples at different concentrations. For this purpose, different volumes of CeO_2−x_@Cit NPs suspensions (nearly 10 mg/mL) were added to 500 μL of the 0.1 mM DPPH solution diluted to 1.5 mL in EtOH, resulting in NP concentrations ranging from 0.13 mg/mL to 0.8 mg/mL. A control experiment was performed by simply diluting 500 μL of the DPPH^·^ solution 0.1 mM to 1.5 mL (Abs_DPPH_t0_). Each mixture was left to react under dark and vigorous stirring at room temperature for 30 min; then the supernatant was recovered by centrifugation, and the UV–vis absorption spectrum was recorded. In particular, the absorption intensity at 517 was monitored for each solution (Abs_DPPH_NPs_) and the % RSA measured based on the following equation:

(1) $$\%\;RSA=\frac{\left({Abs}_{DPPH\cdot \_t0}-{Abs}_{DPPH\cdot \_NPs}\right)}{{Abs}_{DPPH\cdot \_t0}}\times 100$$

A control experiment was carried out using citric acid alone (0.03 mg/mL) in DPPH^·^ assay to assess the contribution of the ligand to the DPPH^·^ scavenging activity of CeO_2_@Cit NPs. The concentration of the citric acid solution was set following the thermogravimetric analysis on CeO_2_@Cit NPs, from which the weight percentage attributed to citric acid was estimated. For CeO_2_@Dopa, a control experiment was performed using polydopamine purposely synthesized by polymerization of dopamine hydrochloride in aqueous solution under alkaline conditions by NaOH (pH = 10). After 48 h, a brown pellet was recovered and used for DPPH^·^ assay at a concentration of 0.01 mg/mL and 0.02 mg/mL.

### 2.7. Evaluation of Cell Viability

The effect of CeO_2−x_@Cit NPs on DITNC1 cell viability was assessed using the MTT assay [3-(4,5-dimethylthiazol-2-yl)-2,5-diphenyltetrazolium bromide] [30]. Briefly, DITNC1 seeded in 96-well plates were treated with CeO_2−x_@Cit (CeO_2−x_@Dopa) NPs at concentrations ranging from 5 to 100 μg/mL. After 24 h of incubation at 37 °C, 5%, CO_2_ cells were washed with PBS and incubated for 2 h at 37 °C and 5% CO_2_ with 0.5 mg/mL of MTT. At the end of the incubation period, the culture medium was discarded, and the formazan crystals formed within the cells were dissolved using absolute ethanol. The concentration of the formazan product was measured by optical absorbance at 545 nm, with 690 nm as the reference wavelength. Cell viability was calculated as a percentage in comparison to the negative control (ctrl), represented by untreated cells, which was set at 100%.

### 2.8. Intracellular Reactive Oxygen Species Detection

The detection of ROS in DITNC 1 was performed as previously described [31]. The cells treated only with DCFH-DA without and with rotenone were used as negative (CTRL) and positive (ROT) controls, respectively. To test the ROS production in the presence of CeO_2−x_ NPs, briefly, cells were pre-treated for 90 min at 37 °C, 5%, CO_2_ with CeO_2−x_@Cit or CeO_2−x_@Dopa NP preparations at concentrations of 10 μg/mL. To assess the intrinsic pro-oxidant or antioxidant effect of CeO_2−x_@Cit and CeO_2−x_@Dopa NP, in a separate set of experiments, cells were pre-treated with the NPs samples as described above and were subsequently loaded with 4 μM DCFH-DA, without rotenone or with 10 μM rotenone. After incubation for 50 min, the supernatants were discarded, and the fluorescence intensity of cells was analyzed via spectrofluorimetric analysis at 525 nm under excitation at 485 nm in a microplate reader. Results were normalized to cell viability, and ROS production was expressed as the relative percentage of photoluminescence (PL) intensity compared to the negative or positive control.

Parametric one-way analysis of variance (ANOVA), followed by Dunnett’s multiple comparison post hoc test, was used to compare ROS levels and cell viability under different experimental conditions. Data were obtained from at least three independent experiments, with each data point representing the mean of triplicate measurements within a single experiment. Data were analyzed by GraphPad Prism 5.0 (GraphPad Software, Inc., San Diego, CA, USA).

### 2.9. Characterization Techinques

TEM characterization was performed using a JEOL JEM 1011 (JEOL, Akishima, Tokyo, Japan) transmission electron microscope operating at 100 kV and equipped with a high-resolution CCD camera. Two microliters of the NP suspension were dropped onto a carbon-coated copper grid, and the solvent was left to evaporate. Statistical analysis of NP sizes in the samples and their respective distributions was conducted using the image analysis software ImageJ 1.54g. For each sample, the average NP size and the relative percentage standard deviation (σ%) were calculated.

The FT-IR characterization was carried out by using a 670 FT-IR spectrometer (Varian, Palo Alto, CA, USA) equipped with a diamond ATR accessory of 2 mm and a deuteratedtryglicine sulfate (DTGS) detector. One microliter of each sample was put on the internal reflection element, and the solvent was allowed to evaporate. Spectra were recorded in the range 4000–400 cm^−1^ acquiring 16 scans with a nominal resolution of 1 cm^−1^.

Thermogravimetric analysis (TGA) was carried out using a Pyris 1-Perkin Elmer instrument under a nitrogen flow of 40 mL/min at the heating rate of 20 °C/min in a temperature range from 50 °C to 700 °C. Thermograms were collected using powder of dried NP samples [32].

UV–vis absorption spectra were recorded using a Cary 5000 (Agilent, Santa Clara, USA) UV/vis/NIR spectrophotometer. The samples were purposely diluting, and spectra in the wavelength range 200–800 nm were recorded.

XPS analyses were performed with a PHI 5000 Versa Probe II spectrometer (Physical Electronics) equipped with a monochromatic Al Kα X-ray source (1486.6 eV), operated at 15 kV and 24.8 W, with a spot size of 100 µm. Survey (0–1200 eV) and high-resolution spectra (C1s, O1s, and Ce3d) were recorded in FAT mode at pass energies of 187.85 and 29.35 eV, respectively. Surface charging was compensated by means of a dual-beam charge neutralization system. All spectra were collected at an angle of 45° with respect to the sample surface. The hydrocarbon component of the C1s spectrum was used as an internal standard for charging correction, and it was fixed at 284.8 eV. Spectra were processed with MultiPak software v. 9.5.0.8 (Physical Electronics). Atomic concentrations were determined from the high-resolution spectra after subtracting a Shirley-type background, using the Scofield sensitivity factors set in the MultiPak software.

The Ce 3d core level splits into two states by spin–orbit interaction, with Ce 3d5/2 and Ce 3d3/2 assigned as bands of v and u type, respectively. The Ce 3d fitted spectra, deconvoluted in ten Gaussian–Lorentzian peaks, were assigned separately to the two ionic cerium species, and the area of each species was calculated as follows:

(2) $${A(Ce}^{3+})=A\left(v^{0}\right)+A\left(v\prime \right)+A\left(u^{0}\right)+A(u\prime )$$

(3) $${A(Ce}^{4+})=A\left(v\right)+A\left(v\prime \prime \right)+A\left(v\prime \prime \prime \right)+A\left(u\right)+A\left(u^{\prime \prime }\right)+A(u^{\prime \prime \prime })$$

The relative concentration of Ce^3+^ and Ce^4+^ ions was calculated as follows:

(4) $$\left[{Ce}^{3+}\right]=\frac{A({Ce}^{3+})}{A\left({Ce}^{3+}\right)+A({Ce}^{4+})}*100$$

(5) $$\left[{Ce}^{4+}\right]=\frac{A({Ce}^{4+})}{A\left({Ce}^{3+}\right)+A({Ce}^{4+})}*100$$

## 3. Results and Discussion

### 3.1. Synthesis and Aqueous Phase Transfer of CeO_2−x_@OAm Nanoparticles via Citrate Ligand Exchange

**Figure 2:**
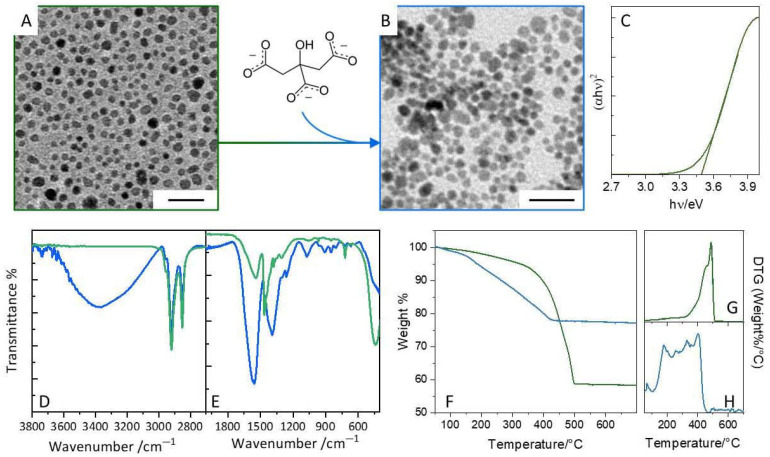
TEM micrographs (**A**,**B**), Tauc plot as determined by applying Kubelka–Munk function (**C**); FT-IR in attenuated total reflection mode (**D**,**E**), thermogravimetric (**F**) and first derivative (**G**–**H**) curves of CeO_2−x_@OAm (green line) and CeO_2−x_@Cit (blue line).

CeO_2−x_ NPs have been synthesized by thermal decomposition of Ce(NO_3_)_3_·6H_2_O in high-boiling ODE in the presence of OAm as a coordinating agent [26]. This method yields ultra-small, monodispersed, and colloidally stable NPs. OAm is expected to bind to the NP surface via amino groups, controlling NP growth, while the exposed long alkyl chain provides steric stabilization, ensuring long-term stability and long-term dispersion in non-polar media. TEM micrograph (Figure 2A) reveals spherical CeO_2−x_@OAm NPs, with an average diameter of ~3 nm (σ% = 21%). Optical characterization shows an energy gap of 3.5 eV, measured by the Tauc plot (Figure 2C). Such a value is significantly larger than that reported for bulk CeO_2_ (3.2 eV) consistent with NPs in with the quantum confinement regime (Bohr radius ~ 3.5 nm). The ultrasmall particle attained through this synthetic procedure is favorable for enhancing antioxidant activity [33]. However, despite the advantages of the synthetic approach in providing ultrasmall and minimally aggregated NPs, their dispersibility in organic solvents demands the development of an effective phase transfer procedure to render them water dispersible and thus suitable for biological systems [34].

Citric acid is an effective ligand to achieve water-dispersible CeO_2−x_ NPs. At physiologic pH, citrate molecules coordinate the CeO_2−x_ NPs surface via multidentate interactions, exposing outside the negatively charged carboxylate groups that confer good colloidal stability in aqueous medium via Coulombic repulsion [35]. To successfully replace the pristine OAm capping layer via ligand exchange, it is essential to choose a solvent or solvent mixture capable of solubilizing both the hydrophobic-capped NPs and the hydrophilic citrate ligands. While Monnier et al. [29] previously used a mixture of chlorobenzene and DMSO for this purpose on Fe_2_O_3_ NPs, in this work chlorobenzene has been replaced for the first time by CME as a more environmentally sustainable alternative. The high boiling point of CME allows the ligand-exchange reaction to be carried out at high temperature (100 °C), which promotes efficient desorption of OAm and anchoring of citrates onto the NP surface. TEM analysis of CeO_2−x_@Cit (Figure 2B) confirms that NPs preserve their original shape and size (Figure S1). Compared to CeO_2−x_@OAm, the citrate-coated NPs appear more closely packed, consistent with the presence of a thinner, less sterically hindered surface layer. Ligand exchange and surface modification have been further validated through FT-IR spectroscopy in ATR mode (Figure 2D–E, see Figure S2) and thermogravimetric analysis (Figure 2F–H) comparing samples before (green line) and after (blue line) the ligand exchange. The FT-IR spectrum of CeO_2−x_@OAm shows intense peaks at 2920 cm^−1^ and 2850 cm^−1^ corresponding to the antisymmetric and symmetric C-H stretching vibrations of the -CH_2_- groups, respectively, along with a weaker signal at 2959 cm^−1^, characteristic of the asymmetric stretching mode of C-H in the methyl group [36,37,38]. A broad band at 1542 cm^−1^ corresponds to stretching modes of oxidized –NH_2_ groups (e.g., nitrile and imine), while a weak shoulder at 1645 cm^−1^ may arise from oleyl chain C=C stretching or the NH_2_ rocking vibration of unbound OAm. Additional features at 1465 cm^−1^ (CH_2_ scissoring), 1378 cm^−1^ (CH_3_ bending), and 1059 cm^−1^ (C-N stretching) further confirm the presence of OAm.

After ligand exchange with citrate, the FT-IR spectrum of CeO_2−x_@Cit NPs displays a dominant broad O–H stretching band between 3200 and 3500 cm^−1^, indicative of hydroxyl groups or adsorbed water, confirming successful surface functionalization with citrate [39,40]. The presence of citrate is also confirmed by the two broad bands at 1560 cm^−1^ and 1390 cm^−1^ [41], which are assigned to the symmetrical and asymmetrical vibrations of the carboxylate groups. Additional peaks at 1255 cm^−1^ and 1069 cm^−1^ correspond to the C-O stretching modes of citrate. Both NPs samples show a Ce-O-C bond at 1060 cm^−1^ and Ce-O bending modes in the 600–400 cm^−1^ region [42,43,44,45]. Characteristic signals of cerium oxide are also present in the 800–880 cm^−1^ range [46].

To further evaluate the nature and amount of ligands on the CeO_2−x_ NP surface, thermogravimetric analysis (TGA, Figure 2F) has been performed, from which first derivative curves (Figure 2G–H) have been plotted. For, CeO_2−x_@OAm NPs a progressive weight loss is observed between 100 and 330 °C, corresponding to the evaporation of free or physically adsorbed OAm. Further weight losses occur in a wide temperature range between 330–500 °C, which are ascribed to OAm bound to the NP surface (330–400 °C) [47] and to the conversion of residual Ce(OH)_3_, on the NP surface, into CeO_2_ at 450 °C and 488 °C [48]. In the case of CeO_2−x_@Cit NPs, the first weight loss occurs between 50° and 100 °C, associated with the evaporation of weakly bound water. A second loss, between 100° and 220 °C, is attributed to the release of more strongly bound intramolecular water. These results are consistent with FT-IR findings, which also indicate the presence of water molecules on the NP surface. The weight loss between 220 and 340 °C can be ascribed to surface-bound citrate molecules, covering a broad temperature range due to hydrogen interactions that influence the thermal behavior of the passivating layer. Additional weight losses at higher temperatures can be ascribed to the conversion of surface cerium hydroxylate groups. Based on thermogravimetric data, the citrate content in the NP sample is estimated to be approximately 10% by weight.

**Figure 3:**
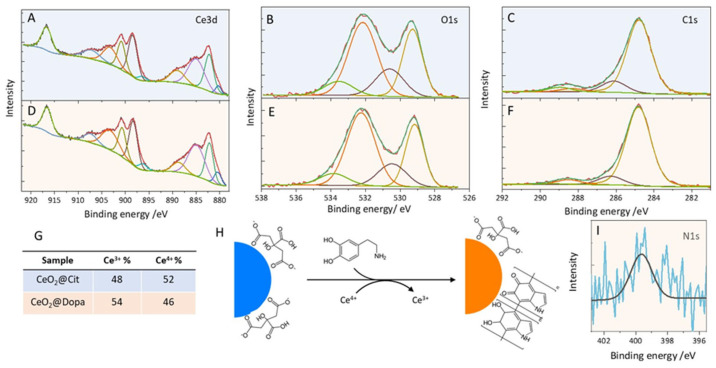
High-resolution XPS spectra Ce3d (**A**,**D**), O1s (**B**,**E**), and C1s (**C**,**F**), N1s (**I**) of CeO_2−x_@Cit sample (**A**–**C**) and CeO_2−x_@Dopa sample (**D**–**F**,**I**). Table of the composition of Ce^3+^ and Ce^4+^ (**G**), as determined from weight ratio of the integral area of Ce^3+^ and Ce^4+^ components. (**H**) Representative scheme of t CeO_2−x_@Cit NPs functionalization with dopamine with a sketch of the surface chemistry as estimated by chemical characterization. Color code: blue for CeO_2−x_@Cit sample and orange for CeO_2−x_@Dopa sample.

XPS investigation has been carried out on CeO_2−x_@Cit NPs and high-resolution spectra of Ce3d, O1s, C1s, and N1s are reported in Figure 3. For materials with mixed +3/+4 cerium valence states, the XPS high-resolution spectrum of Ce3d is complex and comprised of ten peaks. As described by Deshpande et al. [18], based on Burrough’s convention [49], four peaks are associated with Ce^3+^, (v_0_, v′, u_0_, and u′), and six peaks with Ce^4+^ (v, v″, v‴, u, u″, and u‴) [50,51,52,53,54]. The content of 48% and 52% determined for Ce^3+^ and Ce^4+^ (Figure 2G), respectively, highlights a higher Ce^3+^ content than that usually reported for bulk ceria (30% and 70% for Ce^3+^ and Ce^4+^, respectively) [55], confirming that the decrease in CeO_2−x_ particle size is accompanied by reduction of Ce^4+^ to Ce^3+^ to counteract the oxygen loss from the surface.

The O1s high-resolution spectrum (Figure 3B) shows two contributions at 529.3 eV and 530.6 eV, which can be attributed to lattice oxygen in ceria NPs and to the presence of the OH groups on the nanoceria surface, respectively. To get further information on the passivating shell at the NP surface, high-resolution C1s spectrum is acquired and curve-fitted with four contributions (Figure 3C, Table S1) peaked at 284.8, 286.1, 288.3, and 289.0 eV, which are characteristic of C−C or C−H, C−OH and/or C-OC, coordinated carboxylates or carbonyl, and free carboxyl moieties, respectively. Since no signal of nitrogen has been detected in the XPS survey spectrum, complete removal of OAm from the NPs surface and its successful replacement by citrates is confirmed.

### 3.2. Ligand-Exchange with Dopamine and Characterization of CeO_2−x_@Dopa

CeO_2−x_@Dopa NPs have been prepared starting from CeO_2−x_@Cit NPs by treating them with a dilute dopamine hydrochloride solution. Dopamine is reported to spontaneously form a metal complex at the CeO_2−x_ surface, transferring electrons to Ce^4+^ sites, which are then reduced to Ce^3+^, simultaneously oxidizing the catechol structure into quinone structures [16,24,56,57]. Under ambient conditions, dopamine polymerization can also naturally occur. The surface chemistry and the cerium valence state following dopamine treatment have been rationalized by means of XPS characterization. Quantitative analysis of the Ce3d high-resolution spectrum (Figure 3D,G) reveals a slightly larger Ce^3+^ content (54%) in CeO_2−x_@Dopa NPs than CeO_2−x_@Cit NPs (46%), confirming the reducing effect induced by treatment with dopamine. High-resolution C1s and N1s spectra (Figure 3F and 3I, respectively; Table S1) have been examined to understand the chemical nature of the surface-bound species. In the C1s spectrum, a decrease in the peak at 286.1 eV, assigned to C-OH and an increase in the component at nearly 288.3 eV, characteristic of carbonyl moieties (Table S1), support the formation of oxidized *o-*quinones structures bound to the NP surface. Additionally, the decrease in the contribution at nearly 289.0 eV, attributed to COOH, suggests that residual citric acid molecules are mostly in their deprotonated form. Even though π−π* shakeup peaks at nearly 291 eV, specific of the aromatic groups, are not visible, being usually poorly intense, the evidence of nitrogen in the N1s (Figure 3I) high-resolution spectrum, which is not present in the CeO_2−x_@Cit sample, confirms the success of the ligand exchange procedure. The binding energy value of the N1s peak at 399.6 eV suggests the formation of nitrogen-heterocyclic structures such as those expected in dimeric [58] or polymeric structures of dopamine [59]. The occurrence of polymerization is also confirmed by TGA (see Figure S3), that which a prominent weight loss at 440 °C, a temperature higher than those reported for dopamine and dopamine derivatives.

This in-depth characterization opens the venue to a different representation of the surface functionalization of CeO_2−x_ NPs treated with dopamine than compared to previous studies [60,61]. Besides the confirmation of an increase in the Ce^3+^/Ce^4+^ ratio, even though not to a large extent, and the formation of *o-*quinone structures, polymerization of dopamine with nitrogen involved in heterocyclic structures is also proposed [16].

Based on this comprehensive characterization, after dopamine treatment the CeO_2−x_@Dopa NPs feature an inorganic core bearing a size and surface cerium valence ratio similar to CeO_2−x_@Cit, and specific *o-*quinone groups and a polymerized coating. To test the antioxidant activity of CeO_2−x_@Dopa NPs, a comparative approach is used by performing chemical assays and in vitro experiments in a biological environment, using CeO_2−x_@Cit as a control sample.

### 3.3. Catalytic and Radical Scavenging Activity of CeO_2−x_@Cit and CeO_2−x_@Dopa

To preliminarily evaluate the catalytic and radical scavenging activity of the two CeO_2−x_ NPs samples, two in vitro non-enzymatic chemical assays have been performed. These included monitoring the spectral change of the CeO_2−x_ NPs solutions upon treatment with H_2_O_2_ and in the colorimetric DPPH^·^ assay. Even though these non-enzymatic chemical tests do not fully replicate the complex behavior of NPs in a biological environment, they allow a preliminary evaluation of the catalytic activity displayed by each NPs sample.

#### 3.3.1. Catalytic Activity of CeO_2−x_ Nanoparticles Towards H_2_O_2_

H_2_O_2_ is an ROS endogenously produced under oxidative stress conditions, and naturally enzymatically removed by CAT activity or in situ decomposition into ^·^OH species following the Fenton reaction. CeO_2−x_ NPs can mimic the enzymatic reaction and scavenge H_2_O_2_. First, H_2_O_2_ reacts at the surface of CeO_2−x_ NPs, forming cerium-peroxo and hydroperoxo complexes (Ce-O_2_^2−^ and/or Ce-OOH^−^ complexes), further transformed into O_2_ or ^·^OH via reaction mediated by the easy shift between cerium valence states. Formation of these complexes has been reported to be accompanied by a visible color change of the colloidal solution of CeO_2−x_ NPs from yellow to brown and a significant increase in the absorption intensity between 300 and 600 nm [22,62]. This spectral response is commonly exploited to qualitatively assess the interaction of H_2_O_2_ with the CeO_2−x_ NP surface and to compare the catalytic activity across different CeO_2−x_ NP samples [21]. Finocchiaro et al. [62] demonstrated that adsorption of H_2_O_2_ is size-dependent, with smaller CeO_2−x_ NPs (e.g., 4.5 nm) exhibiting enhanced absorption compared to the larger ones (7.8 nm, 23 nm, and 28 nm) upon addition of H_2_O_2_ at increasing concentrations. The higher reactivity shown by the smaller NPs is ascribed to the high fraction of Ce^3+^ relative to the larger ones. Surface coating also plays a critical role: hydrophobic or bulky ligands can hinder H_2_O_2_ diffusion towards CeO_2−x_ NPs active surface sites, thereby limiting the formation of reactive cerium-peroxo or hydroperoxo intermediates [21,62].

**Figure 4:**
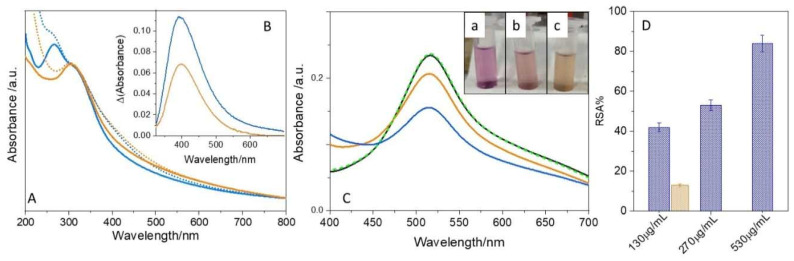
(**A**) UV–vis absorption spectra of CeO_2−x_@Cit (blue line) and CeO_2−x_@Dopa (orange lines) samples before (solid line) and after (dashed line) addition of H_2_O_2_. (**B**) Absorption peak obtained after subtracting the absorbance from dispersion devoid of H_2_O_2_ for CeO_2−x_@Cit (blue line) and CeO_2−x_@Dopa (orange line) samples. (**C**) Test of the scavenging activity of DPPH·: Vis absorption spectra of DPPH^·^ before (black trace) and after addition of citric acid (0.01 mg/mL, dashed green trace), CeO_2−x_@Cit (0.13 mg/mL blue line) and CeO_2−x_@Dopa (0.13 mg/mL orange line) samples. Inset, pictures of the DPPH^·^ solutions after 30 minutes in the dark without (a)and with CeO_2−x_@Dopa (b) and with CeO_2−x_@Cit (c). (**D**) Bar graph of the percentage of DPPH· radical scavenging activity (RSA%) of CeO_2−x_@Cit at increasing dose (blue bars) and CeO_2−x_@Dopa (0.13 mg/mL orange bar).

To evaluate this, UV–vis absorption spectra of untreated and H_2_O_2_-treated CeO_2−x_@Cit and CeO_2−x_@Dopa NPs have been recorded (Figure 4A). Both untreated samples show the characteristic profile of CeO_2−x_ NPs with an absorption shoulder at 300 nm attributed to Ce 5d–O 2p valence states. CeO_2−x_@Cit sample displays an additional band at 265 nm, attributed to surface-bound hydrated species, which disappear after dopamine treatment. Furthermore, CeO_2−x_@Dopa shows an increased intensity of the absorption tail in the 300–500 nm range, consistent with the characteristic spectral signature of *o*-quinone groups, in line with the XPS findings [63,64]. Upon treatment with H_2_O_2_ both samples display increased absorption below 270 nm, due to free H_2_O_2_, and enhanced absorption above 300 nm [65].

By subtracting the absorbance of the suspensions devoid of H_2_O_2_ (Figure 4B) from the absorbance of the H_2_O_2_-treated samples, an absorption peak centered at 390 nm is observed, which can be attributed to cerium-peroxide and cerium-hydroperoxide species generated due to adsorption of H_2_O_2_ at the particle surface [15,21,62].

Notably, CeO_2−x_@Cit NPs exhibit a stronger absorption peak than CeO_2−x_@Dopa, suggesting higher H_2_O_2_ interaction and, hence, greater intermediate formation. Since both samples share similar size and Ce^3+^/Ce^4+^ ratios, as assessed through chemical and morphological characterization, the different reactivity towards H_2_O_2_ can be attributed to surface chemistry. CeO_2−x_@Cit NPs, capped with a small hydrophilic citrate coating, permits easier H_2_O_2_ diffusion and surface interaction. In contrast, the dense hydrophobic polymeric coating on CeO_2−x_@Dopa NPs hinders H_2_O_2_, limiting the formation of peroxo or hydroperoxo species at the NP surface and hence reactivity [62].

#### 3.3.2. Assessment of DPPH^·^ Scavenging Activity

The colorimetric DPPH^·^ assay is a well-established, widely employed protocol to evaluate the antioxidant capacity of natural and synthetic molecules or nanoantioxidant materials, based on monitoring the bleaching of the absorption band of the stable radical DPPH^·^, centered at nearly 516 nm, due to its conversion into DPPH_2_ by electron and proton transfer [66]. CeO_2−x_ NPs have demonstrated the ability to scavenge DPPH [67]. Compared to the redox potential of the couple Ce^4+^_(aq)_/Ce^3+^_(aq)_ (1.71 V versus SHE), CeO_2−x_ NPs exhibit a significantly lower redox potential of nearly 0.2 V (versus SHE at pH 7) [67,68], which makes them capable of reducing the DPPH^·^/DPPH_2_ couple (0.77 V versus SHE). Electrons are supplied by the cerium states switching between Ce^+4^ and Ce^+3^ and protons by metal oxide surface hydroxyl groups, ligands, or solvent. However, the surface coating on CeO_2−x_ NPs can influence this process, either hampering electron transfer through steric hindrance [21] or enhancing the reduction of DPPH^·^ by providing protons or due to intrinsic antioxidant properties [48].

As shown in Figure 4C, exposure to both CeO_2−x_@Cit and CeO_2−x_@Dopa NPs results in a noticeable color change of the DPPH^·^ solution from violet to pale yellow (Figure 4C(a–c)) alongside a decrease in absorbance at 516 nm after 30 min. At a concentration of 130 μg/mL, CeO_2−x_@Cit displays markedly higher radical scavenging activity (42%) than CeO_2−x_@Dopa (13%) at 33 μM DPPH^·^. Control experiments have been carried out using citrate alone to determine whether the radical scavenging activity could be partially attributed to surface-bound ligands. A citrate solution at a concentration equivalent to that estimated to be on CeO_2−x_@Cit NPs based on TGA analysis has been tested in the DPPH^·^ assay. This citrate solution showed minimal reduction in DPPH^·^ absorption after 30 min, suggesting a negligible DPPH^·^ scavenging activity of citrate at the tested concentration, being not relevant in the CeO_2−x_@Cit scavenging behavior. In the case of CeO_2−x_@Dopa NPs, control experiments with independently synthesized polydopamine present a significant challenge, as it is difficult to replicate the specific functionalities of the polymer coating formed in the presence of CeO_2−x_. This is due to electron transfer between dopamine or its polymerized derivatives and CeO_2−x_ surface, which alters the chemical structure of the resulting PDA layer. When polydopamine is, synthesized separately in alkaline aqueous solution without CeO_2−x_@Cit NPs, it predominantly features catechol groups (Figure S2). These catechol-rich structures are known to exhibit different reactivity compared to quinone-containing systems [6,25]. Tested in the DPPH^·^ assay, the synthesized polydopamine, despite its catechol-dominated composition, shows negligible DPPH^·^ scavenging activity, at the concentration equivalent to that estimated on the CeO_2−x_@Dopa surface. A modest increase in scavenging (up to ~7%) is observed only when the PDA concentration is doubled (Figure S4). These results, consistent with previous reports [25], suggest that the negligible DPPH^·^ scavenging activity of CeO_2−x_@Dopa can be attributed to their sample surface functionalization [25,69], characterized by the presence of *o*-quinone, and possibly to the hampered accessibility of DPPH^·^ to the ceria core, hindered by the sterically protective PDA coating [70].

Based on these observations, CeO_2−x_@Dopa appear less reactive toward H_2_O_2_ and in radical scavenging DPPH^·^ compared to CeO_2−x_@Cit. This reduced activity is likely due to the polymeric coating, which may limit radical species diffusion to the NP surface—an essential step preliminary to electron transfer. It is worth noting that the DPPH^·^ assay, despite its usefulness for quick screening, does not accurately reflect the complex interactions occurring with biologically relevant radicals. It also does not capture antioxidant effects, such as inhibition of autoxidation of organic substrates or other cellular processes. Therefore, to fully evaluate the potential offered by these nanoantioxidants, additional in vitro experiments within cells are required.

#### 3.3.3. Antioxidant Effect of CeO_2−x_@Cit and CeO_2−x_@Dopa on DITNC1 Cells

Astrocytes play a crucial role in maintaining redox homeostasis in the brain, making them particularly relevant for assessing the neuroprotective potential of antioxidant compounds [71]. In this study, the DITNC1 cell line, represented by immortalized astrocytes derived from rat brain tissue, has been selected to evaluate the antioxidant power effects of CeO_2−x_ NPs since it represents a valuable model for investigating oxidative stress in the central nervous system. Cell cultures have been treated with CeO_2−x_ NPs at a concentration of 10 μg/mL that falls within the non-cytotoxic range (Figure S5). The production of ROS under both basal and oxidative stress conditions, induced by the addition of rotenone, was monitored using the DCF-DA assay. Even though DCFH-DA lacks selectivity in ROS detection, it has been reported as an easily and widely available procedure, relatively inexpensive, and easy to load into cells.

Rotenone is a potent inhibitor of mitochondrial Complex I, a critical enzyme involved in the electron transport chain, and has been widely used to study mitochondrial-induced oxidative stress [72]. Indeed, the inhibition of Complex I causes an accumulation of electrons within the mitochondrial matrix, which can react with O_2_ to produce ROS, particularly ^·^O_2_^−^. Because of the increased ROS levels, cell components, including lipids, proteins, and DNA, are damaged, contributing to mitochondrial dysfunction and cellular oxidative stress.

**Figure 5:**
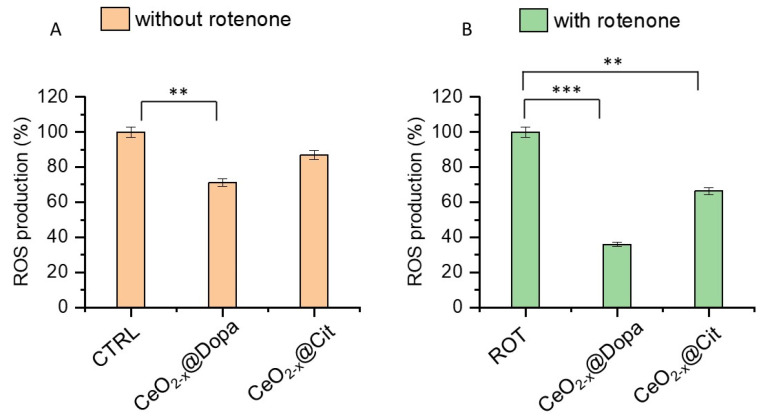
ROS production in DITNC1 cells treated with CeO_2−x_@Cit or CeO_2−x_@Dopa. The presence of ROS assessed by measuring the changes of the fluorescent signal of DCFH-DA. ROS production expressed as percentage (%) of photoluminescence (PL) intensity in comparison to negative (CTRL) (**A**) or positive control (ROT) (**B**) represented by unstimulated and untreated cells in serum-free medium (CTRL) and rotenone treated cells (ROT), respectively. Bar graphs in panel A and panel B referred to cells treated with or without rotenone, respectively, also in the presence of CeO_2−x_@Cit or CeO_2−x_@Dopa. The bar value represents the mean value ± Standard deviation of three independent experiments performed on different cell populations. Statistically significant decrease in comparison to CTRL or ROT indicated by asterisks (one-way ANOVA followed by Dunnet’s post hoc test was used to compare ROS levels and cell viability under different experimental conditions ** *p* < 0.01, *** *p* < 0.001. Data were obtained from at least three independent experiments, with each data point representing the mean of triplicate measurements within a single experiment.).

The incubation of DITNC1 with each CeO_2−x_ NPs preparation, under basal conditions, resulted in a statistically significant decrease in ROS production for both CeO_2−x_@Cit (10%) and CeO_2−x_@Dopa (28%) compared to the corresponding control (Figure 5A). Treatment with rotenone resulted in a 70% increase in ROS production in DITNC1 cells compared to the negative control, confirming the role of this compound as an inducer of oxidative stress. The ROS scavenging activity shown by CeO_2−x_ NPs enhanced under oxidative stressed conditions (Figure 5B) induced by the addition of rotenone. In this case the decrease in ROS production, in comparison to ROT control, was 34% and 65% for CeO_2−x_@Cit and CeO_2−x_@Dopa, respectively. A greater ability to scavenge ROS is experienced by the cells incubated with CeO_2−x_@Dopa, both without and with addition of rotenone, rather than CeO_2−x_@Cit.

*Discussion.* The evaluation of ROS production within DITNC1 cells without and with the addition of rotenone confirms the intrinsic antioxidant activity of CeO_2−x_ NPs. Even though according to literature studies [4,15,73], the enhanced antioxidant activity shown by CeO_2−x_ NPs treated with dopamine might be attributed to the higher Ce^3+^/Ce^4+^ ratio, quantification of cerium valence ratio by XPS has highlighted only a slightly different Ce^3+^/Ce^4+^ ratio between CeO_2−x_@Cit and CeO_2−x_@Dopa, whose extent could not support the high efficacy in ROS scavenging shown by CeO_2−x_@Dopa.

Thanks to an extensive characterization of the NPs, which offered a clear picture of the surface functional groups, and thanks to fundamental knowledge built on solid literature studies [6,25,58], we proposed that the reason behind the better performance in limiting ROS production in biological environments demonstrated by CeO_2−x_@Dopa compared to CeO_2−x_@Cit, should not be only attributed to the intrinsic properties of CeO_2−x_ NPs but should also be sought in the reactivity associated with the functional groups at the surface of the NPs. In particular, the presence of a surface coating bearing *o*-quinone structures must be considered. Although several studies described the improvement in antioxidant activity offered by citrates in citrate-functionalized NPs [74,75], our results indicate that *o*-quinone functionalization is more effective in boosting the catalytic activity of CeO_2−x_ NPs in biological environments.

**Scheme 1:**
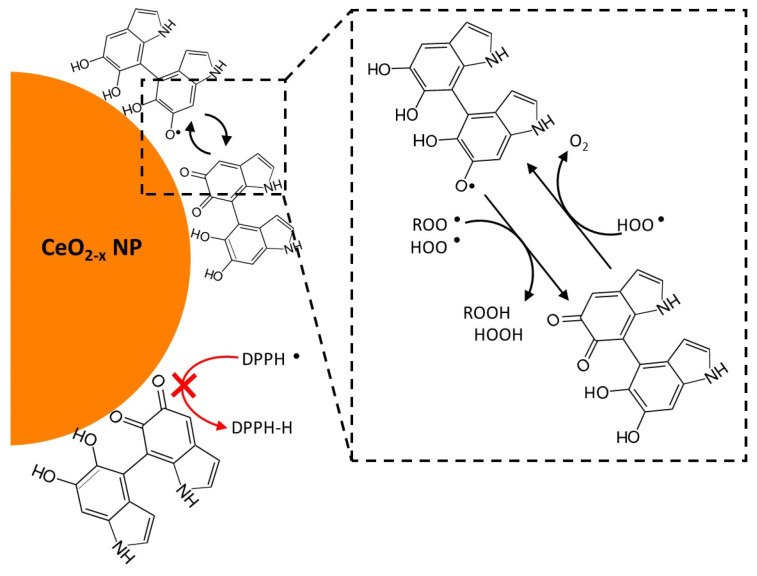
Representative scheme of the hydroperoxyl radical scavenging activity and organic substrate autoxidation chain breaking promoted by CeO_2−x_ NPs functionalized with *o-*quinone structures generated after treatment with dopamine.

As illustrated in Scheme 1 and demonstrated by Amorati et al. for polydopamine [25,58,69], *o*-quinones can scavenge HOO^·^ radicals by oxidizing them to O_2_ while being reduced to o-semiquinones. These intermediates can then interrupt the autoxidation chain of organic substrates by donating electrons and protons to ROO^·^, thereby regenerating the *o*-quinone form. This redox cycling adds a complementary mechanism to the intrinsic activity of CeO_2−x_ NPs, enhancing chain-breaking antioxidant capacity and suppressing the propagation of ROS production induced by autoxidation of organic substrates.

The improved ROS scavenging activity observed for CeO_2−x_@Dopa arises from these complex redox mechanisms that are not captured by the DPPH^·^ assay. In the latter, the antioxidant activity appears underestimated due to the limited diffusion of the DPPH^·^ radical toward the NPs surface, which is hindered by the surface coating, and the lack of reactivity between *o*-quinone and DPPH^·^.

The comparative experimental approach employed, using CeO_2−x_@Cit NPs as a control to evaluate the redox behavior of our CeO_2−x_@Dopa NPs, and the detailed characterization of both samples, focusing on their chemical states and functional groups, along with well-established literature knowledge of the antioxidant properties of *o-*quinone functionalities and CeO_2−x_ NPs, provided a robust structure–function correlation. The comparative framework offered meaningful insight into the enhanced antioxidant performance of CeO_2−x_@Dopa. Our findings highlight the potential of engineered nanostructures to interfere with oxidative stress pathways and suppress oxidative chain reactions more effectively. Indeed, this work, being clearly a material-oriented study, does not present an exhaustive mechanistic investigation of the antioxidant behavior, even though solid background supports the discussion of the achieved outcomes and conclusion.

## 4. Conclusions

This study has confirmed the pivotal role of CeO_2−x_ NPs in quenching ROS and its effective enhancement by NP surface engineering. Water-dispersible CeO_2−x_@Dopa NPs have been synthesized using a colloidal approach involving thermal decomposition of a precursor in hot coordination solvent, yielding ultrasmall NPs approximately 3 nm in diameter, followed by phase transfer by ligand-exchange reaction with citric acid and post-synthetic treatment with dopamine hydrochloride. A more sustainable ligand-exchange procedure has been exploited, using CME with DMSO as the reaction medium. Comprehensive characterization has revealed two key findings: (i) CeO_2−x_ NPs showed an increased Ce^3+^/Ce^4+^ ratio compared to bulk CeO_2_ (Ce^3+^/Ce^4+^ 30%/70%), attributed to their nanoscale; (ii) post-synthetic treatment with dopamine resulted in a slight increase in Ce^3+^ and a surface coating characterized by a polymeric structure bearing *o-*quinone structures. Evaluation of the radical scavenging activity—both via chemical non-enzymatic assays and in vitro experiments within DITNC1 cells—has highlighted the crucial role played by surface chemistry in the material’s antioxidant behavior. Despite the diffusion of DPPH^·^ and H_2_O_2_ on the CeO_2−x_@Dopa surface being partially hindered by the polymeric coating, the overall ROS scavenging activity in the biological environment has increased. Unlike earlier studies that attributed antioxidant enhancement solely to a higher Ce^3+^/Ce^4+^ ratio, our comparative analysis between CeO_2−x_@Cit and CeO_2−x_@Dopa suggested that the increased ROS scavenging ability can be traced back to the presence in the latter sample of the *o-*quinone surface functionalities. These groups, whose presence in the polymer coating of CeO_2−x_@Dopa has been demonstrated by complementary UV–vis absorption, FT-IR, and XPS characterization, can contribute, by quenching HOO^·^ radicals, to disrupting the autoxidation chain of organic substrate, a key process in the propagation of oxidative stress. This finding underscores the strategic value of dopamine treatment, which synergistically amplifies the NPs antioxidant capacity of CeO_2−x_, offering an innovative approach for applications requiring high antioxidant activity in complex biological settings.

The mechanistic insights presented in this study can open the venue for future in-depth investigations. As demonstrated by Amorati et al., the use of well-defined chemical systems—such as those able to generate hydroperoxyl radical in situ and promote the autoxidation of organic substrates—offers a valuable strategy for evaluating the antioxidant activity of CeO_2−x_@Dopa compared to CeO_2−x_@Cit, particularly through kinetic monitoring of oxygen consumption. In parallel, fluorescence microscopy investigation of properly treated cells could provide high spatial resolution and sensitivity, enabling real-time visualization of ROS production and identification of intracellular compartments involved. Furthermore, for any potential biomedical application, it will be essential to assess NPs colloidal stability in physiological media as well as their interaction with biological systems, including protein corona formation, biodistribution, and clearance, prior to advancing towards in vivo studies and clinical translation.

## Data Availability

Dataset available on request from the authors.

## Supplementary Materials

The following supporting information can be downloaded at: https://www.mdpi.com/article/10.3390/antiox14080916/s1, Figure S1: size distribution obtained by TEM of CeO_2−x_ NPs before and after ligand-exchange with citric acid; Table S1: Sketches of the two water-dispersible sample of CeO_2−x_@Cit and CeO_2−x_@Dopa and peak area of the different contributions of the XPS high resolution C1s spectrum; Figure S2: FT-IR of CeO_2−x_@OAm, CeO_2−x_@Cit, CeO_2−x_@Dopa and polydopamine; Figure S3: TGA of CeO_2−x_@Dopa; Figure S4: Test of the scavenging activity of DPPH^·^ with polydopamine at different concentrations; Figure S5: cell viability test of CeO_2−x_@Cit and CeO_2−x_@Dopa.
