# Supplementary material for: Enhancing Antioxidants Performance of Ceria Nanoparticles in Biological Environment via Surface Engineering with o-Quinone Functionalities
Source: Antioxidants (Basel). 2025 Jul 25;14(8):916. doi: 10.3390/antiox14080916 (PMC12382933; doi:10.3390/antiox14080916)
Supplement: Supplementary file 1 [file antioxidants-14-00916-s001.zip › antioxidants-3755932-supplementary.pdf]

Supplementary Material

Enhancing Antioxidants Performance of Ceria Nanoparticles in Biological Environment via Surface Engineering with *o*-quinone Functionalities

Pierluigi Lasala[1], Tiziana Latronico[2], Umberto Mattia[1], Rosa Maria Matteucci[3,4], Antonella Milella[1], Matteo Grattieri[1,4,5], Grazia Maria Liuzzi[2], Giuseppe Petrosillo[6], Annamaria Panniello[4], Nicoletta Depalo[4,5], Maria Lucia Curri[1, 4,5], Elisabetta Fanizza[1,4,5]

[1] *Chemistry Department, University of Bari, via Orabona 4 70126 Bari, Italy*

[2] *Department of Bioscience, Biotechnology and Environment, University of Bari, Via Orabona 4, 70126 (Bari) Italy*

[3] *Polytechnic University of Bari, Via Orabona 4, Bari 70125, Italy*

[4] *CNR-Institute for Chemical Physical Process (IPCF), via Orabona 4 70126 Bari, Italy*

[5] *National Interuniversity Consortium of Materials Science and Technology, INSTM, Bari Research Unit, 70126, Bari, Italy*

[6] *CNR-Institute of Biomembranes, Bioenergetics and Molecular Biotechnologies (IBIOM), Via Giovanni Amendola, 122/O 70126, Bari, Italy, giuseppe.petrosillo@cnr.it*

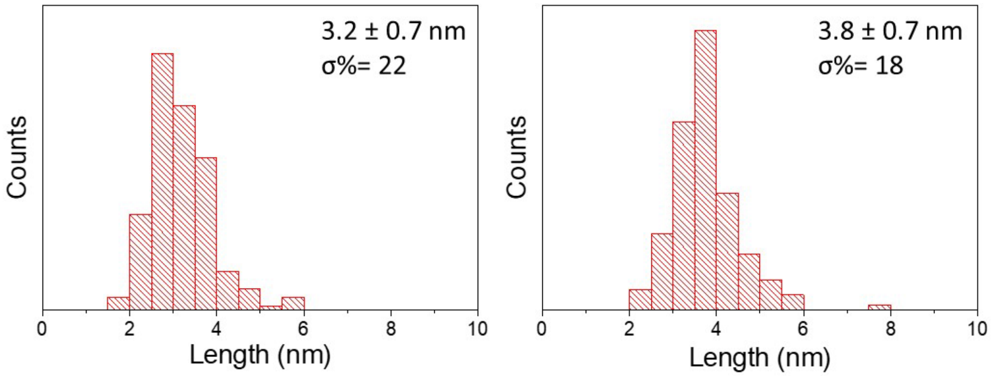

Figure S1. Size distribution of the sample CeO_{2-x}@OAm (A) and CeO_{2-x}@Cit (B).

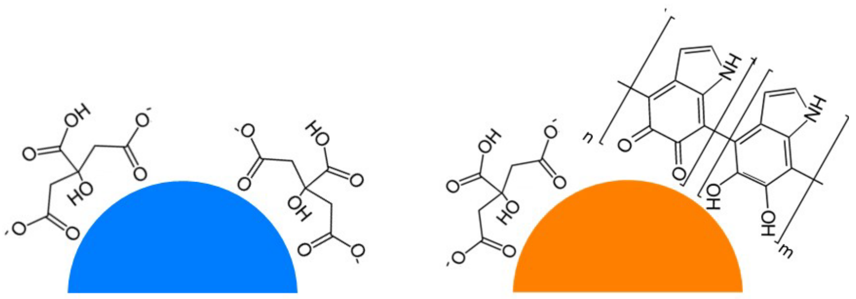

Binding energy/eV	*Area %* *CeO_{2-x}@Cit*	*Area %* *CeO_{2-x}@Dopa*
284.8 (C−C or C−H)	79.0	84.2
286.1 C−OH or C-OC	12.2	9.3
288.3 (COO^- or C=O)	3.8	5.5
289.0 (COOH)	5.0	0.9

Table S1. Sketches of the CeO_{2-x}@Cit (left) and CeO_{2-x}@Dopa (right) and table reporting the binding energy values of the peaks used to fit the C1s high resolution XPS spectra of each sample and their relative % area.

In Figure S2A the FT-IR spectra in attenuated total reflectance (ATR) mode of the CeO_{2-x}@Olam, (green line) CeO_{2-x}@Cit (blue line) and CeO_{2-x}@Dopa (orange line) across a wide wavelength range are reported.

In Figures S2B and S2C the same spectra have been zoomed in on in the wavenumber range 1750–1300 cm^{-1} and 1300-750 cm^{-1}, respectively, and reported along with that of polydopamine synthesized in aqueous alkaline condition for comparison. In particular, the FT-IR characterization of the CeO_{2-x}@Dopa in panel B (orange line) shows two intense bands that could be presumably ascribed to asymmetric and symmetric stretching of carboxylate from residual citrates along with stretching modes of C=C of the aromatic ring and vibration of -C=O of the quinone. In particular, the quinone structure usually presents a peak centered within 1750-1650 cm^{-1}, which can corroborate the shift to higher wavenumber and broadening of the band in the range 1700-1550 cm^{-1} (band centered at 1580 cm^{-1} in CeO_{2-x}@Dopa and 1560 cm^{-1} in CeO_{2-x}@Cit).

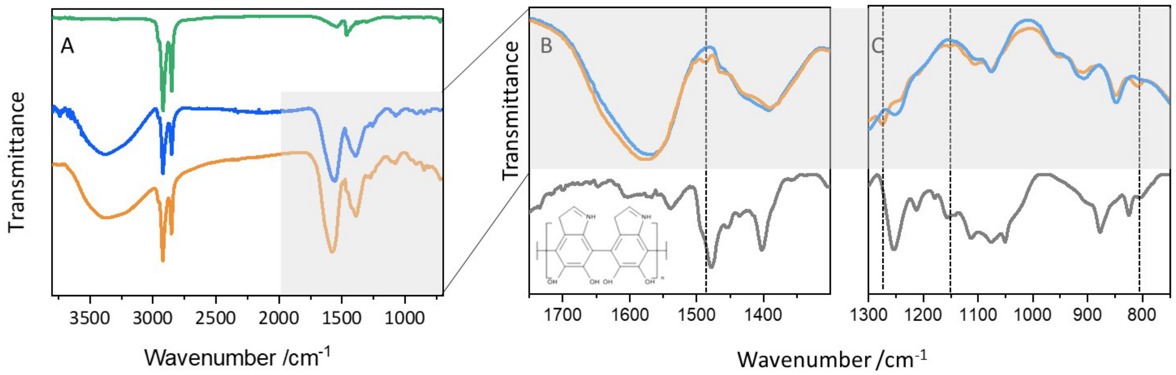

Figure S2. FT-IR spectra in attenuated total reflection mode (A) in wavenumber range 3800–700 cm^{-1} zoom-on in 1750–1300 cm^{-1} (B) and 1300–750 cm^{-1} of CeO_{2-x}@Olam (orange line), CeO_{2-x}@Cit (blue line), CeO_{2-x}@Dopa (orange line), and polydopamine synthesized by polymerization of dopamine hydrochloride in alkaline aqueous solution (grey line).

Furthermore, a hyperfine structure in the FT-IR spectra of the CeO_{2-x}@Dopa with features like those displayed in the PDA spectrum has been detected. The peaks at 1488 cm^{-1}, at 1273 cm^{-1}, and at 1153 cm^{-1} in CeO_{2-x}@Dopa can be attributed to the stretching modes of C=C of the aromatic ring, to the -C-O bending of catechol structures, and to the C=N stretching of the heteroaromatic structure. In particular, peaks at 1488 cm^{-1}, at 1273 cm^{-1} are shifted to higher wavenumbers and are less intense than those observed in PDA as a consequence of coordination to the NPs surface and change of the electronic environment surrounding the corresponding functional groups.

In Figure S3, thermogravimetric analysis of the CeO_{2-x}@Dopa confirms the change in surface chemistry. The first derivative curve shows a prominent weight loss at 440 °C, which can be attributed to polydopamine, along with a continuous weight loss at lower temperatures that could be presumably ascribed to residual citrate molecules.

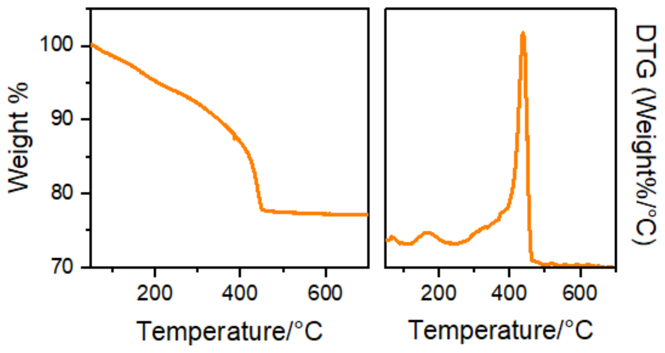

Figure S3. Thermogravimetric analysis of CeO_{2-x}@Dopa.

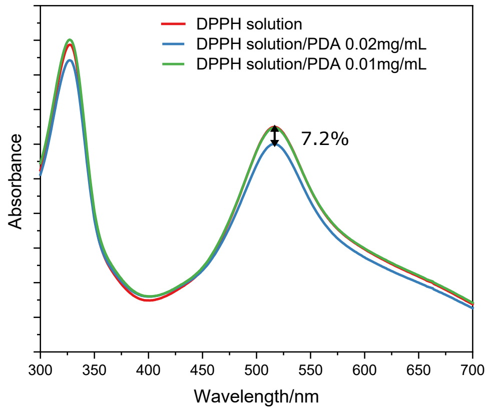

Figure S4. Test of the scavenging activity of DPPH˙: UV–vis absorption spectra of DPPH˙ before (red trace) and after addition of PDA (0.01 mg/mL, green trace), PDA (0.02 mg/mL blue line).

The biocompatibility of CeO_{2-c}@Cit and CeO_{2-x}@Dopa NPs has been assessed on the DITNC1 cell line by the MTT assay. As shown in Figure S3, the tested preparations did not affect cell viability across the entire concentration range of NPs investigated (5−100 µg/mL) after 24 hours of treatment.

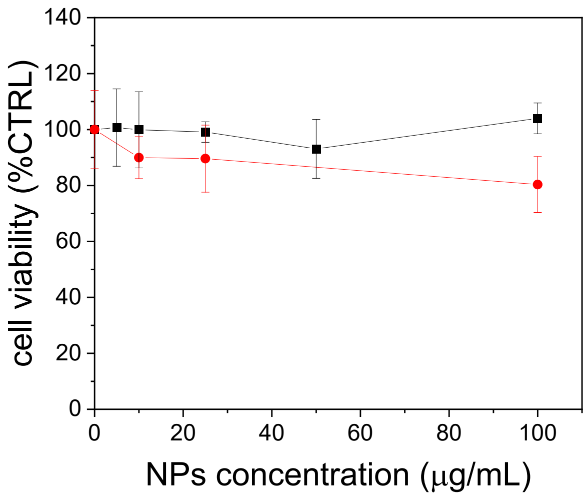

Figure S5. Cell viability assay for the CeO_{2-x}@Cit (black line) and CeO_{2-x}@Dopa (red line) samples.
